# Recent Advances in the Theory of Vision

**Published:** 1872-10

**Authors:** 


### Recent Advances in the Theory of Vision. By H. Helmholtz.

The present number completes the translation, by Dr. F. W. Abbott, of the valuable Essay of H. Helmholtz upon the recent advances in the Theory of Vision. It is to be regretted that the paper could not have all appeared in a single number, and as it is so complete, it should be furnished to the profession in book or pamphlet form, but the members of the profession who take deepest interest in it are, perhaps, too few to warrant the outlay. We have no doubt but the readers of the Journal have read with deep interest the installments of the paper as published; certainly, all who appreciate its worth will thank Dr. Abbott for his translation. We are personally under many obligations for the opportunity of publishing so valuable a translation in our journal.

